# An anti-infective synthetic peptide with dual antimicrobial and immunomodulatory activities

**DOI:** 10.1038/srep35465

**Published:** 2016-11-02

**Authors:** O. N. Silva, C. de la Fuente-Núñez, E. F. Haney, I. C. M. Fensterseifer, S. M. Ribeiro, W. F. Porto, P. Brown, C. Faria-Junior, T. M. B. Rezende, S. E. Moreno, T. K. Lu, R. E. W. Hancock, O. L. Franco

**Affiliations:** 1 Departamento de Biologia, Instituto de Ciências Biológicas, Programa de pós-graduação em Genética e Biotecnologia, Universidade Federal de Juiz de Fora, Juiz de Fora–MG, Brazil; 2 S-Inova Biotech, Pós-graduação em Biotecnologia, Universidade Católica Dom Bosco, Campo Grande, MS, Brazil; 3 Synthetic Biology Group, MIT Synthetic Biology Center, Massachusetts Institute of Technology, Cambridge, Massachusetts, United States of America; 4 Research Laboratory of Electronics, Massachusetts Institute of Technology, Cambridge, Massachusetts, United States of America; 5 Department of Biological Engineering, and Department of Electrical Engineering and Computer Science, Massachusetts Institute of Technology, Cambridge, Massachusetts, United States of America; 6 Broad Institute of MIT and Harvard, Cambridge, Massachusetts, United States of America; 7 Harvard Biophysics Program, Harvard University, Boston, Massachusetts, United States of America; 8 The Center for Microbiome Informatics and Therapeutics, Cambridge, Massachusetts, United States of America; 9 Department of Microbiology and Immunology, University of British Columbia, Vancouver, British Columbia, Canada; 10 Centro de Análises Proteômicas e Bioquímicas, Programa de Pós-Graduação em Ciências Genômicas e Biotecnologia, Universidade Católica de Brasília, Brazil; 11 Programa de Pós-graduação em Patologia Molecular, Universidade de Brasília, Brasília, Brazil; 12 Department of Chemical Engineering, Massachusetts Institute of Technology, Cambridge, Massachusetts, United States of America; 13 Lacen, Laboratório Central de Saúde Pública do Distrito Federal, Brasília, DF, Brazil; 14 Curso de Odontologia, Universidade Católica de Brasília, Brazil; 15 Pós-graduação em Ciências da Saúde, Universidade de Brasília, Brazil

## Abstract

Antibiotic-resistant infections are predicted to kill 10 million people per year by 2050, costing the global economy $100 trillion. Therefore, there is an urgent need to develop alternative technologies. We have engineered a synthetic peptide called clavanin-MO, derived from a marine tunicate antimicrobial peptide, which exhibits potent antimicrobial and immunomodulatory properties both *in vitro* and *in vivo*. The peptide effectively killed a panel of representative bacterial strains, including multidrug-resistant hospital isolates. Antimicrobial activity of the peptide was demonstrated in animal models, reducing bacterial counts by six orders of magnitude, and contributing to infection clearance. In addition, clavanin-MO was capable of modulating innate immunity by stimulating leukocyte recruitment to the site of infection, and production of immune mediators GM-CSF, IFN-γ and MCP-1, while suppressing an excessive and potentially harmful inflammatory response by increasing synthesis of anti-inflammatory cytokines such as IL-10 and repressing the levels of pro-inflammatory cytokines IL-12 and TNF-α. Finally, treatment with the peptide protected mice against otherwise lethal infections caused by both Gram-negative and -positive drug-resistant strains. The peptide presented here directly kills bacteria and further helps resolve infections through its immune modulatory properties. Peptide anti-infective therapeutics with combined antimicrobial and immunomodulatory properties represent a new approach to treat antibiotic-resistant infections.

Antibiotics are often considered the most successful intervention in modern human medicine. However, their efficacy has been hampered by the rise of drug-resistant bacteria^1^. In addition, despite the marked increase in efforts to find new drugs, no new antibiotics have been licensed for clinical use in recent years^1^. The urgent need to develop novel antimicrobial therapeutics has recently been emphasized by the estimation that by the year 2050, 10 million people will die annually worldwide as a direct result of antimicrobial resistance^2^. Antimicrobial peptides (AMPs) represent a potential alternative to available antibiotics^3^,^4^. These peptides are evolutionarily conserved molecules that serve in host defence against microbial infections. Present in all living organisms, these peptides generally range in size from 12 to 50 amino acids and are cationic due to the presence of lysine and arginine residues^5^. Moreover, about 50% of the amino acids in AMPs are hydrophobic and they can adopt amphipathic structures, which enables them to interact with and penetrate cell membranes^3^,^5^. Naturally occurring peptides as well as synthetic derivatives have been shown to exhibit potent antimicrobial, anti-biofilm and immunomodulatory properties^6^,^7^,^8^. The multifunctional nature of these peptides makes them attractive for therapeutic use, as targeting multiple cellular pathways in living bacteria is known to reduce the selective pressure for the development of resistance.

Clavanin A (VFQFLGKIIHHVGNFVHGFSHVF-NH_2_) is a naturally occurring AMP that was first isolated in 1997 from the hemocytes of the marine tunicate *Styela clava*^9^,^10^. In addition to its cationic and amphipathic characteristics, this peptide possesses an amino acid sequence rich in histidine, phenylalanine and glycine residues. Clavanin A has been shown to be broadly effective *in vitro* and *in vivo* against Gram-negative and Gram-positive bacteria as well as fungi. This peptide also has the advantageous property over other AMPs of being active even at high salt concentrations and in acidic pH^10^,^11^,^12^,^13^,^14^.

Here, we first confirmed the broad-spectrum antimicrobial activity of clavanin A and next found that clavanin A also exhibited immunomodulatory properties^12^. We decided to engineer clavanin A in order to improve its antimicrobial and immune modulation capabilities. For this, first we computationally searched for a conserved region in AMPs that was predicted to exhibit both antibacterial and immunomodulatory activities. We found a hit in the conserved oligopeptide FLPII, which was found to be present in a large number of peptides with immunomodulatory activity. Interestingly, this hydrophobic region is conserved in plant lipocalins, a family of proteins that are known to interact with different types of membranes and organelles through this short hydrophobic oligopeptide domain. We next incorporated the hydrophobic region composed of five non-polar amino acid residues FLPII to the N-terminal end of clavanin A, and called the new molecule clavanin-MO (FLPIIVFQFLGKIIHHVGNFVHGFSHVF-NH_2_). In minimal inhibitory concentration and flow cell microfluidic assays this novel synthetic peptide generally exhibited increased antimicrobial activity compared to its wild-type predecessor peptide, presented a range of immune modulatory activities both *in vitro* and *in vivo*, and conferred protection against lethal infections caused by Gram-negative and Gram-positive drug-resistant pathogens in animal models. Additionally, the peptide lacked cytotoxicity both *in vitro* and *in vivo* against erythrocytes, monocytes and fibroblasts. The peptide therapy described here represents a new and innovative approach to treat infectious diseases through its combined antimicrobial and immunomodulatory properties.

## Results

### Antibacterial activity profile of peptides

In the present study, a hydrophobic amino acid oligopeptide tag FLPII was added to the N-terminus of the natural AMP clavanin A to generate a modified clavanin sequence called clavanin-MO with increased overall hydrophobicity (Table S1). The antibacterial activity profiles of clavanin A and clavanin-MO are shown in Table 1. Both clavanin A and clavanin-MO were active against all bacterial strains tested, with MIC values ranging from 1.5 to 45 μM (Table 1). Interestingly, in all cases, clavanin-MO showed improved antimicrobial activity (MICs 2-to-5 fold lower) compared to clavanin A. It was observed that *S. aureus* ATCC 29213 and *E. coli* ATCC 8739 were the least susceptible to the action of clavanin-MO (MICs of 24 and 12 μM, respectively) while *B. subtilis* ATCC 6633 and *E. faecalis* ATCC 12953 were the most susceptible (MIC of 1.5 μM) (Table 1). In addition, the antibacterial activity of clavanin-MO was superior to that of the human cathelicidin AMP LL-37 against both Gram-negative and Gram-positive bacteria. Importantly, clavanin-MO was more effective at killing antibiotic-resistant Gram-negative strains, such as KPC positive strains of *E. coli* and *K. pneumoniae* or multidrug resistant *E. coli* 2101123, compared to the commonly used antibiotics gentamicin (an aminoglycoside) and imipenem (a β-lactam) (Table 1). Finally, clavanin-MO displayed antibacterial activity against methicillin-resistant *S. aureus* clinical isolates thus highlighting the potential of this synthetic peptide to overcome antibiotic resistance.

In parallel studies, we tested the ability of the peptides to eradicate surface-associated biofilms, which are bacterial aggregates that are up to 1000-fold more resistant to antibiotics compared to their planktonic counterparts and are associated with two-thirds of all infections in humans^6^. To assess the anti-biofilm eradication activity of clavanin-MO, we first performed microtiter static biofilm assays and observed increased anti-biofilm activity for clavanin-MO cf. clavanin-A (data not shown). To confirm the biofilm inhibitory potential of clavanin-MO in using a more sophisticated biofilm model, we performed microfluidic flow cell assays as previously described^6^,^15^. Biofilms formed by antibiotic-resistant strains *K. pneumoniae* KPC971 and *S. aureus* MRSA were grown for 2 days, after which they were treated with peptides for 24 hours. At a peptide concentration of 16 μg.ml^−1^ (~5 μM), clavanin-MO did not fully eradicate either biofilm (Fig. S1), however it did decrease the overall biofilm biomass of drug-resistant strain *K. pneumoniae* KPC971 while *S. aureus* MRSA biofilms were relatively unaffected (Fig. S1). Since previous reports identified the stress response nucleotide ppGpp-mediated pathway as a target for some synthetic peptides with anti-biofilm activity^6^,^16^, we asked whether clavanin A or clavanin-MO were affecting this cellular pathway. In this context, we performed thin layer chromatography assays of nucleotides extracted from intact *P. aeruginosa* PAO1 and *S. aureus* HG001 bacteria in the absence and presence of increasing concentrations of peptide (Fig. S2). As expected, the peptides did not reduce ppGpp levels in either *P. aeruginosa* PAO1 or *S. aureus* HG001 (Fig. S2), even when tested to peptide concentrations as high as 64 μg.mL^−1^.

### Structural characterization studies

Next, we investigated the behaviour of both clavanin A and its synthetic variant clavanin-MO in a DOPC bilayer by means of molecular dynamics (Fig. S3). Since it is known that clavanin A adopts an α-helical conformation in DOPC bilayers^17^, we reasoned that such simulations would provide insights into any potential conformational differences between clavanin A and clavanin-MO. The evolution of the backbone root-mean-square deviation (RMSD) during the simulation indicated that the structures were maintained, since the RMSD were above 4 Å (Fig. S1B). Clavanin-MO showed more fluctuations in RMSD than clavanin A, however both peptides behaved similarly in the DOPC bilayers, whereby after 100 ns the structures suffered an inclination relative to the Z axis, reflecting their being more embedded in the membrane (Fig. S1A). From the results obtained in these studies, we conclude that the addition of the five residues FLPII enhanced the affinity of clavanin-MO towards the hydrophobic core of membranes. Overall, both peptides behaved similarly in contact with membranes thus indicating that the increased antimicrobial activity displayed by (Table 1) is not due to its direct interaction with membranes.

### *In vitro* and *in vivo* toxicity studies

**Figure 1:**
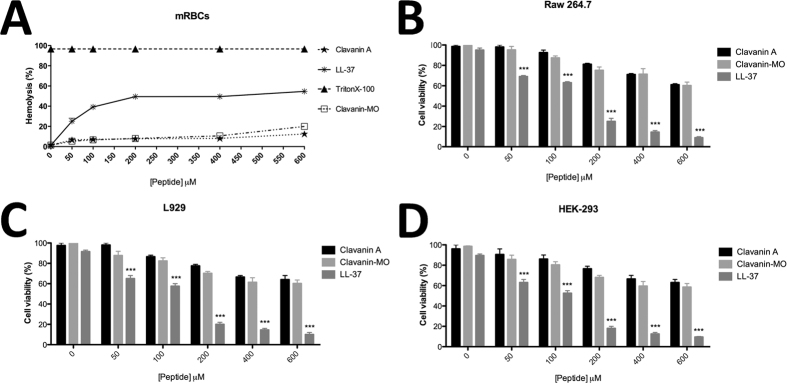
Lack of peptide toxicity. Assays evaluating the cytotoxic activity of the peptides against fresh mouse red blood cells (mRBCs) 1% Triton X-100 (triangle) served as positive control (100% hemolysis). (**A**) The release of hemoglobin was measured at 540 nm. In assays evaluating the cytotoxic activity of the different peptides and antibiotics against RAW 264.7 monocytes (**B**) L929 (**C**) and HEK-293 (**D**) cells were incubated for 24 h. cell viability was assessed by MTT assay. Values within the table correspond to the concentration (given in μM) of the Clavanin A (star), clavanin-MO (square) and LL-37 (asterisk). Data represent the mean of three experiments performed in triplicate. Data are expressed as mean ± standard deviation. *p < 0.05; **p < 0.01; ***p < 0.001. Statistical analysis was performed using a Student’s t test with Bonferroni correction for multiple testing.

Next, we determined the cytotoxic effects of peptides clavanin A and clavanin-MO on mouse red blood cells (mRBCs), RAW264.7 macrophage, L929 mouse fibroblast cell lines and human embryonic kidney cells 293 (HEK-293). Cell viability, through detection of cellular oxidative metabolism, was measured using the MTT assay after 24 hours of treatment with different peptide concentrations. Similar to previous studies, clavanin A showed no haemolytic activity and no significant cytotoxic effects on cells in culture^9^,^12^ (Fig. 1). Addition of the oligopeptide tag did not result in increased toxicity, as clavanin-MO exhibited similar levels of low toxicity as its parent peptide (Fig. 1A) and did not cause cytotoxicity towards RAW264.7, L929 cells and HEK-293 cells at the concentrations required for antimicrobial and immunomodulatory activity (Fig. 1B–D, respectively). Consistent with these *in vitro* results, neither clavanin A nor clavanin-MO caused any apparent toxicity in mice treated intraperitoneally with a dose as high as 50 mg.kg^−1^ of peptide (data not shown), five times the maximum utilized here for successful anti-infective therapy.

### *In vitro* immunomodulatory properties

**Figure 2:**
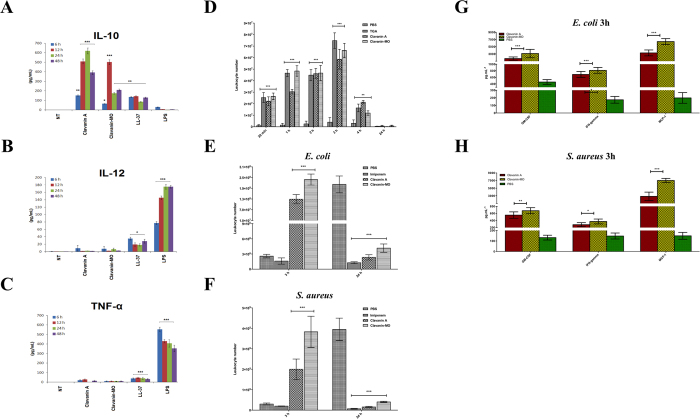
Immunomodulatory activity: Peptide-mediated modulation of innate immunity responses. *In vitro* assays. Effect of clavanin A and clavanin-MO on cytokine release (pg.mL^−1^) from RAW264.7 macrophages. RAW264.7 cells were stimulated with LPS (10 ng.mL^−1^) and treated with 2 μM of peptides. Cathelicidin LL-37 (1 μM) was used as control. All experiments were performed in triplicate. Pro-inflammatory cytokine levels for (**A**) IL-10, (**B**) IL-12p70 and (**C**) TNF-α were analyzed for samples collected 6, 12, 24 and 48 hours after treatment. Data are expressed as mean ± standard deviation. *p < 0.05; **p < 0.01; ***p < 0.001. Statistical analysis was performed using Bonferroni correction, where was compared NT group with the other groups. *In vivo* assays. Clavanin A and clavanin-MO induced migration of leukocytes into the peritoneal cavity of mice. Leukocyte migration was evaluated into the peritoneal cavity of uninfected C57BL/6 mice 30 min, 1, 2, 3, 4, 6 and 24 hours after treatment with 10 mg.kg^−1^ of peptides (**D**). Thioglycolate (TGA) 3% was used as a positive control for leukocyte migration. Leukoyte recruitment to the peritoneal cavity was also assessed in mice infected with *E. coli* ATCC 8739 (**E**) or *S. aureus* ATCC 29213 (**F**). Leukocytes were counted 3 and 24 hours after infection and treatment with 10 mg.kg^−1^ of peptide, 10 mg.kg^-1^ of imipenem or PBS control. Data were expressed as mean ± standard deviation. Statistical analysis was performed using Fisher’s exact test. *p < 0.05; **p < 0.01; ***p < 0.001. Effects of clavanin A and clavanin-MO on the release of cytokines into the peritoneal cavity of mice infected with *E. coli* ATCC 8739 or *S. aureus* ATCC 29213. Cytokine release into the peritoneal cavity of C57BL/6 mice was measured by ELISA after 3 hours of peptide administration for mice infected with *E. coli* ATCC 8739 and treated with 10 mg.kg^−1^ clavanin A or clavanin-MO (**G**). A separate group of mice were infected with *S. aureus* ATCC 29213, treated with 10 mg.kg^−1^ of clavanin A or clavanin-MO, and peritoneal cytokine profiles were measured after 3 hours of administering peptides (**H**). PBS served as a negative control for these experiments. Data are expressed as mean ± standard deviation. *p < 0.05; **p < 0.01; ***p < 0.001. Statistical analysis was performed using Fisher’s exact test.

Certain cationic peptides are capable of modulating the innate immune response due to their ability to stimulate the induction of chemokines while suppressing potentially harmful pro-inflammatory cytokines^16^,^18^,^19^,^20^. Here, we evaluated the anti-inflammatory activities of peptides clavanin A and clavanin-MO on murine macrophage-like RAW 264.7 cells stimulated with 10 ng.ml^−1^ LPS (Fig. 2A–C) and compared them to the anti-endotoxin effect of the human cathelicidin peptide LL-37. RAW 264.7 cells stimulated with LPS alone produced low levels of IL-10 while strongly inducing IL-12 and TNF-α levels. In the presence of peptides, there was a significant increase in IL-10 production, particularly in groups treated with clavanin A and clavanin-MO compared to groups exposed to LL-37 (Fig. 2A). All three peptides caused a significant and almost complete reduction in the release of pro-inflammatory cytokines IL-12 and TNF-α in LPS-stimulated cells (Fig. 2B,C, respectively).

### *In vivo* peptide-mediated immune modulation

#### Peptides are chemotactic for leukocytes in mice

An important immunomodulatory activity exerted by some peptides is their ability to stimulate the migration of leukocytes to the site of an infection, as this is a key step into resolving infections 20,21,22. Therefore, we examined whether clavanin A and clavanin-MO were capable of inducing the migration of leukocytes to the infection focus and if these peptides could promote leukocyte recruitment into the intraperitoneal cavity of healthy mice and mice infected with either *E. coli* ATCC 8739 or *S. aureus* ATCC 29213. As seen in Fig. 2D, there was a substantial and significant peptide-induced increase in the number of leukocytes in the peritoneal fluid of healthy animals, similar to leukocyte migration induced by positive controls groups treated with thioglycolate (TGA). In mice not treated with peptide and infected with *E. coli* ATCC 8739 (Fig. 2E) and *S. aureus* ATCC 29213 (Fig. 2F) there was a large increase in leukocyte counts in the peritoneal cavity 24 hours post infection, likely reflecting the establishment of the infection in mice. In peptide treated groups, the peak of leukocyte migration was observed much earlier, only 3 h post-infection in both healthy and infected mice (Fig. 2D–F). Interestingly, leukocyte numbers were significantly reduced in peptide treated mice 24 hours post-infection, similar to levels seen in mice treated with the β-lactam antibiotic imipenem (Fig. 2E,F). This can be attributed to the resolution of the bacterial infection in mice treated with peptides clavanin A and clavanin-MO, consistent with our bacterial cell count results (Fig. 2I–L).

#### Peptides modulate innate immunity in mice

We then tested the ability of the peptides to modulate the immune response in mice. In an early infection phase (i.e., 3 hours post-infection), both clavanin A and clavanin-MO significantly triggered the release of cytokines GM-CSF, IFN-γ and MCP-1 in mice infected with both *E. coli* and *S. aureus* (Fig. 2G,H).

### Efficacy of peptides in bacterial infection models

#### Synthetic peptide clavanin-MO provided broad-spectrum protection against lethal infections caused by antibiotic-resistant pathogens in mice

**Figure 3:**
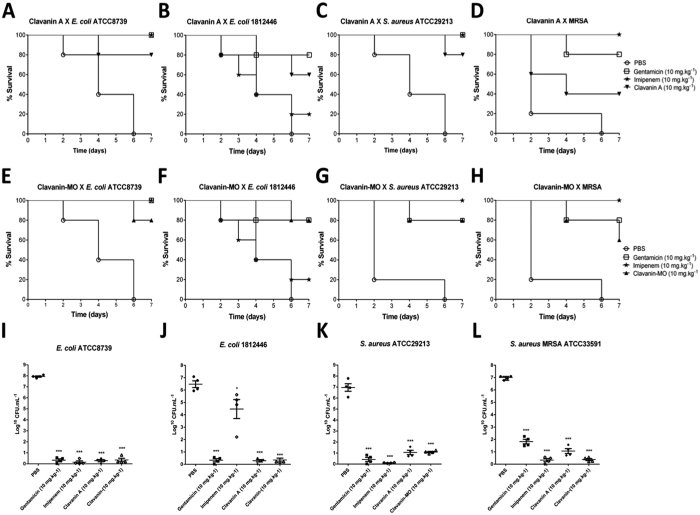
Peptide efficacy in bacterial infection models. Mice were infected with ~2 × 10^7^ CFU of *E. coli* ATCC 8739 (**A**,**E**) or *E. coli* KPC-positive ID N°.1812446 (**B F**) or ~2 × 10^9^ CFU of *S. aureus* ATCC 29213 (**C**,**G**) or *S. aureus* MRSA ATCC 33591 (**D**,**H**). Mice were treated i.p. with a single dose of clavanin **A** 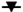, clavanin-MO 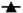, gentamicin 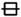, imipenem 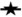 (10 mg.kg^−1^) and PBS 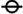 3 h after infection. Survival of mice was evaluated over time during an 8-day period. Evaluation of the protective activity of the peptides in murine models of infection with ~2 × 10^7^ CFU of *E. coli* ATCC 8739 (**I**) or *E. coli* KPC-positive ID N°1812446 (**J**) or~2 × 10^9^ CFU of *S. aureus* ATCC 29213 (**K**) or *S. aureus* (MRSA) ATCC 33591 (**L**). Mice were treated i.p. with a single dose of peptide (10 mg.kg^−1^) 3 hours after infection. The bacterial load was measured by counting bacteria in the peritoneal lavage 24 hours after infection. Data are expressed as mean ± standard deviation. *p < 0.05; **p < 0.01; ***p < 0.001. Statistical significance of differences between experimental groups of animals was determined using the Fisher’s exact test.

Due to the potent and very promising antimicrobial and immunomodulatory properties of clavanin-MO, we decided to test its anti-infective potential. We established a mouse model of aggressive bacterial infection similar to previously described^12^,^13^. Mice were infected with one of the following bacteria via intraperitoneal (i.p.) injection: *E. coli* ATCC 8739, *E. coli* KPC-positive ID N°. 1812446, *S. aureus* ATCC 29213 or *S. aureus* (MRSA) ATCC 33591 and 3 hours later treated i.p. with 10 mg.kg^−1^ of peptide or antibiotic suspended in sterile saline. As shown in Fig. 3, both clavanin A and clavanin-MO prolonged the survival of infected mice. After 8 days of infection, 80% survival was observed for animals infected with wild-type *E. coli* ATCC 8739 (Fig. 3A) or *S. aureus* ATCC 29213 (Fig. 3C) and treated with 10 mg.kg^−1^ of clavanin A. The same survival rate was seen for mice infected with *E. coli* ATCC 8739 (Fig. 3E), *E. coli* KPC-positive (Fig. 3F) or *S. aureus* ATCC 29213 (Fig. 3G) and treated with 10 mg.kg^−1^ of clavanin-MO. Mice infected with MRSA and treated with clavanin-MO showed 60% survival (Fig. 3H), a 20% increase in survival rate compared to animals treated with its parent peptide clavanin A (Fig. 3D). Twenty-four hours post-infection, peritoneal fluid was collected and bacterial colony-forming unit (CFU) counts were performed. These assays showed a significant decrease in the number of viable bacterial counts in peptide-treated groups (Fig. 3I–L), thus suggesting that the highly protective activity of clavanin A and clavanin MO was due to their bacterial killing activity. In mice infected with drug-resistant *E. coli* (Fig. 3J) peptide treatment led to ~4 orders of magnitude greater bacterial killing compared to the conventional β-lactam antibiotic imipenem.

Overall, peptide treatment led to an effective reduction in both bacterial load and infection-associated mortality.

## Discussion

We have identified a novel synthetic peptide, clavanin-MO, which can directly kill bacteria and simultaneously modulate innate immune responses, thereby providing effective treatment of a broad spectrum of infections, while controlling an excessive inflammatory response that could lead to sepsis. This is in contrast with most conventional antibiotics, which actually stimulate the release of bacterial pathogen-associated signature molecule components and can contribute to the risk of damaging inflammation and sepsis^23^.

Clavanin-MO was generated by adding a hydrophobic oligopeptide sequence FLPII to the N-terminus of the parent peptide clavanin A. This hydrophobic region is conserved in plant lipocalins, which are a family of proteins that are known to interact with different types of membranes and organelles through this short hydrophobic domain (FLPII)^24^,^25^,^26^. Based on our results, we propose that this hydrophobic sequence could be enhancing the affinity of clavanin-MO towards bacterial membranes, resulting in improved antimicrobial activity. In our studies, we did not find substantial differences in the interactions of the peptides with membranes (Fig. S1), therefore indicating that clavanin-MO may kill bacteria through other mechanisms. Generally, it is known that increasing the hydrophobicity of peptides tends to increase their toxicity^27^,^28^,^29^. However, here we have shown that clavanin-MO, despite its increased hydrophobicity, was not toxic *in vitro* or *in vivo*.

In addition, the peptides influenced the inflammatory response of RAW 264.7 cells through the production of anti-inflammatory mediators such as cytokine IL-10 and suppression of pro-inflammatory cytokines such as TNF-α and they significantly stimulated the recruitment of leukocytes to the site of infection *in vivo*. The ability of the peptides to skew host responses to favour cellular recruitment whilst controlling excessive harmful inflammation makes these peptides ideal candidates for treating acute infections and disease conditions such as sepsis. Early stage sepsis is characterized by a hyper-inflammatory state, which results in impaired immune function and eventual immunosuppression^30^. Clavanin-MO could help to balance these delicate host responses to reduce the prolonged production of pro-inflammatory mediators but also enhance the activity and recruitment of leukocytes, which are important for baseline immune function. In this context, it is worth noting that other peptides have been shown to modulate the host immune response. For example, the human cathelicidin LL-37 is known to exert several immunomodulation activities, including recruitment of monocytes *in vivo*^31^,^32^. Other synthetic peptides with immunomodulatory properties (e.g., IDR-HH2, IDR-1002, and IDR-1018) have been shown to induce neutrophil migration and chemokine production, and suppress the release of ROS and pro-inflammatory cytokine TNF-α^22^,^30^,^31^,^32^,^33^.

Importantly, treatment with both clavanin A and clavanin-MO protected mice from lethal infections caused by both Gram-negative and Gram-positive pathogens. Clavanin A was previously shown to promote survival of mice infected with *E. coli* ATCC 8739 and *S. aureus* ATCC 29213^12^,^13^. In the present study we further demonstrated that clavanin-MO exhibited similar protective activity to clavanin A in infections caused by wild-type bacteria. However, clavanin-MO exhibited 20% increased survival cf. clavanin A in mice infected with multidrug resistant *E. coli* and MRSA. Furthermore, the peptides almost completely eradicated the infection after 24 h as revealed by CFU counts from bacteria present in the peritoneal cavity (Fig. 3I–L).

### Significance

The proportion of bacterial infections due to multiply resistant organisms, such as methicillin-resistant *S. aureus* and *E. coli* isolates resistant to last-resort antibiotics such as carbapenems and colistin, continues to increase. At the same time, there has been a decline in the number of antimicrobial therapies under development or approved for human use. This has led to the estimate that by 2050, 10 million people will die per year as a result of antibiotic-resistant infections. Therefore, there is an urgent need to develop novel, alternative approaches to treat infectious diseases. Here, we have identified a single peptide that acts by 1) directly killing bacterial pathogens and 2) targeting the host through stimulation of immune cells and suppression of potentially harmful inflammation that could lead to sepsis. In animal models, peptide treatment protected against lethal infections caused by drug-resistant *E. coli* and *S. aureus* clinical isolates, therefore proving the potential of this peptide therapy as a novel anti-infective to counter the rise of antibiotic resistance. However, further studies are needed to assess the pharmacokinetics and pharmacodynamics of the peptide. Additional strategies to increase the stability of the peptides and their overall therapeutic potential may include incorporation of D-amino acid residues into the peptide sequence.

## Experimental Procedures

### Peptides

Clavanin A, clavanin-MO and cathelicidin LL-37 (Table S1) were synthesized by Shanghai Hanhong Chemical (R.P. of China) using the solid-phase with the N-9-fluorenylmethyloxycarbonyl (Fmoc) strategy and purified by high-performance liquid chromatography (HPLC) (GE, USA)^10^,^34^. The identity of each peptide was confirmed by MALDI ToF MS (Bruker, Germany). Peptide purity used in biologic assays was higher than 95%.

### Strains and growth conditions

Strains used included clinical isolates *Escherichia coli* KPC-positive ID N°.1812446^35^*, Escherichia coli* multiresistant ID N°.2101123^35^ and carbapenemase-producing *Klebsiella pneumoniae* 1825971 (KPC971), as well as reference strains *Bacillus subtilis* ATCC 6633, *Enterococcus faecalis* ATCC 12953, *Staphylococcus aureus* ATCC 29213, Methicillin-Resistant *Staphylococcus aureus* ATCC 33591, *Streptococcus pyogenes* ATCC 19615, *Escherichia coli* ATCC 8739, *Klebsiella pneumoniae* ATCC 13885, *Proteus mirabilis* ATCC 25933, *Pseudomonas aeruginosa* ATCC 15442 and *Salmonella enterica* ATCC 14028. Bacteria were plated on brain heart infusion (BHI) agar (Himedia, India) from a frozen stock. Following 24 h incubation, three isolated colonies were transferred to 1 mL of BHI broth. The broth culture was incubated overnight (12–16 h) at 37 °C with shaking^36^.

### Minimum Inhibitory Concentration (MIC) Determination

Minimum inhibitory concentration (MIC) of peptides and antibiotics was evaluated using the broth microdilution technique in BHI with an initial inoculum of 5 × 10^5^ cells in non-treated polystyrene microtiter plates (Corning, USA) as described by Wiegand *et al*.^37^. The MICs were interpreted as the lowest concentration of peptide or antibiotic that completely inhibited the visible growth of bacteria after 12 h of incubation at 37 °C. Each agent was tested in triplicate in at least three independent experiments^12^.

### Antibiofilm assays

Experiments were performed as described previously^6^,^15^. Briefly, flow cell chambers (dimensions of 1 × 4 × 40 mm) were inoculated with bacteria by injecting 400 μl of an overnight culture. After inoculation, the chambers were left without flow for 2 hours to enable initial bacterial adherence. Biofilms were grown in BM2 glucose minimal medium flowing through the cells at a constant rate of 2.4 ml/h for 48 hours at 37 °C. The matured biofilms were then exposed to clavanin-MO or media alone for an additional 24 hours. Biofilm cells were stained using the LIVE/DEAD BacLight Bacterial Viability kit (Molecular Probes, USA) prior to microscopy experiments. Microscopy was performed using a confocal laser-scanning microscope (Olympus, Fluoview FV1000, USA), and 3D reconstructions were generated using the Imaris software package (Bitplane AG).

### Thin Layer Chromatography

Thin layer chromatography (TLC) assays were used to measure ppGpp levels in biofilms as described previously^6^,^15^. Briefly, untreated or bacteria treated with the peptides were labelled with 10 μCi/ml ^32^P for 3 hours. Samples were then extracted with frozen 13 M formic acid by three cycles of freeze-thaw. Aliquots (7.5 μl) of the supernatants were applied to 20 × 20 cm PEI cellulose TLC plates and resolved with 1.5 M KH_2_PO_4_ (pH 3.4) for 4 hours. After chromatography, nucleotides were visualized by autoradiography and quantified with MolecularImager FX PhosphorImager and Quantity One software (Bio-Rad, USA).

### Cell cultures

Murine macrophage-like cells RAW 264.7 and human embryonic kidney cells 293 (HEK-293) were purchased from the Rio de Janeiro Cell Bank and were maintained in supplemented Dulbecco’s modified Eagle medium (DMEM) (Invitrogen, USA) (4 mM L-glutamine, 10% fetal bovine serum (FBS), 2 mM nonessential amino acids, 50 mg.mL^−1^, gentamicin, and 100 units.mL^−1^, penicillin/streptomycin) in the presence of 5% CO_2_ at 37 °C. Cells were stimulated by adding 0.1 ng/mL LPS (*E. coli* 0111:B4) (Sigma, USA) into the medium specified above. Peptides were added at a concentration of 2 μM were 30 min after addition of LPS. After 6, 12, 24 and 48 h of incubation, plates were centrifuged for 6 min at 400×  g and the supernatants were collected and kept frozen at −20 °C until used for analysis of IL-10, IL-12p70 and TNF-α production by ELISA (see below for detailed experimental procedure)^12^,^38^.

### Hemolysis assays

The hemolytic activity of clavanin A and clavanin-MO was evaluated against fresh mouse red blood cells (mRBCs) by measuring the peptide-induced change in the optical density (OD) at 540 nm (Victor X, Perkin-Elmer, Germany)^39^ of a mRBC cell suspension [a 20% (v/v) erythrocytes/phosphate buffered saline (PBS) suspension]. Maximum lysis (100%) was determined by analyzing the supernatant of erythrocytes that had been incubated with 1% Triton X-100 while PBS was used as a negative control^12^,^39^,^40^,^41^.

### Cytotoxicity assays

Cellular cytotoxicity was measured by a colorimetric assay that makes use of thiazolyl blue tetrazolium bromide (MTT: Sigma, USA). A sub-confluent monolayer culture of L929, RAW264.7 and Human Embryonic Kidney HEK-293 cells were collected by scraper in supplemented DMEM (Invitrogen, USA). Cells were seeded in 96-well microtiter plates at a density of 1.0 × 10^5^ cells per well, with different concentrations of peptide or antibiotic controls (1–600 μM). Cells were incubated at 37 °C in the presence of 5% CO_2_ for 48 h. Following incubation, MTT was added to the cells (10 μL at 5 mg.mL^−1^). The plate was incubated for 4 hours in the presence of 5% CO2 at 37 °C. Formazan crystals was dissolved by the addition of 100 μL of 100% DMSO (Mallinckrodt Chemical, USA) per well. Plates were then gently swirled for 5 min at room temperature to dissolve the precipitate. Absorbance was monitored at 575 nm using a microplate spectrophotometer (Victor X, PerkinElmer, Germany)^12^,^37^,^38^. Maximum cytotoxicity (100%) was determined by cells incubated with 1% Triton X-100, PBS was used as a negative control.

### Animal experiments

Six-week-old female C57BL/6 mice (Central Bioterium of the USP/Ribeirão Preto) were used for all studies. Mice used in this study were housed in an animal facility at the Catholic University of Brasilia (UCB). Mice were provided standard food and water *ad libitum*.

### Ethics Statement

The use of mice was conducted in accordance with the regulations set forward by the respective national animal protection committees and in accordance with European Community Directive 86/609 and the U.S. Association for Laboratory Animal Care recommendations for the care and use of laboratory animals. All the techniques/procedures have been refined to provide for maximum comfort/minimal stress to the animals. Experiments performed have been approved by Animal Ethics Committees of the Catholic University of Brasilia (AECs/UCB), number 005/13.

### *In vivo* toxicity assays

The *in vivo* toxicity of clavanin A and clavanin-MO were evaluated by intraperitoneal (i.p.) injection of each peptide to groups of 10 C57BL/6 mice as described^42^. Each mouse was injected with a 0.5-ml solution of freshly prepared peptide in PBS. Doses of peptide administered per mouse were 0, 10, 30, 50, 70 and 90 mg.kg^−1^ of body weight. Animals were directly inspected for adverse effects after 6 hours, and mortality was monitored for 7 days thereafter^12^,^42^.

### Murine systemic infection model

Based on preliminary experiments to determine an effective challenge bacterial inoculum [*E. coli* ATCC 8739 and *E. coli* KPC-positive ID N°.1812446 or *S. aureus* ATCC 29213 and *S. aureus* (MRSA) ATCC 33591] that resulted in consistent systemic infection without rapidly killing the mice (data not shown), mid-log-phase bacteria were diluted to ~2 × 10^7^ CFU/mouse in PBS for Gram-negative bacteria and ~2 × 10^9^ CFU/mouse in PBS for Gram-positive bacteria^12^,^13^,^35^. Mice were challenged by i.p. injection with 200 μL of bacteria suspended in PBS. The day of challenge was designated as day 1 of the experiment. Three hours after bacterial injection, mice (n = 10) were treated by i.p. injection with 10 mg.kg^−1^ of peptides, gentamicin, imipenem or PBS for 8 days and treated every 24 hours^12^,^43^. For bacterial load evaluation, 5 mice/group were humanely killed 24 h after bacterial administration. To measure bacterial colonization, peritoneal lavage was obtained by washing the cavity with 5 ml of sterile PBS. Samples of these washes were serially diluted and 100 μl of each dilution was spread in duplicate on appropriate agar plates to count colonies^44^.

### Isolation of leukocytes from the peritoneal cavity of mice

C57BL/6 mice were injected i.p. with 10 mg.kg^−1^ of peptide suspended in sterile saline. Mice were euthanized, and peritoneal lavage was carried out at different times post-injection. Additionally, leukocyte counts present in the in peritoneal cavity of mice infected with *E. coli* ATCC 8739 or *S. aureus* ATCC 29213 were determined. Animals infected and treated with peptides were euthanized 3 and 24 hours after treatment, and peritoneal lavage was performed to count leukocytes cells in the peritoneal cavity^45^. Animals were sacrificed, and cells present in the peritoneal cavity were harvested by introducing 3.0 ml of PBS containing 1 mM EDTA. Total cell counts were performed with a cell counter (Coulter AC T series analyzer), and differential cell counts were conducted on cytocentrifuge slides (Cytospin 3; Thermo Shandon) stained by the May-Grünwald-Giemsa (Rosenfeld) method. The results are expressed as the number of cells per cavity^46^.

### ELISA

Cultured cells or peritoneal lavage samples were centrifuged at 1000 × g for 10 min to obtain cell-free samples and stored at −20 °C. Cytokine levels were measured by ELISA using anti-mouse IL-10, IL-12p70 and TNF-α (Peprotech, USA). Cytokine levels were analysed according to the manufacturer’s instructions^35^.

### Molecular modelling studies

Initially, the clavanin-MO sequence was submitted to QUARK *ab initio* modelling server^47^ in order to obtain a full folded clavanin-MO structure. Then, the *ab initio* structure was used together with the NMR structure of clavanin A^14^ as templates for construction of the final model of clavanin-MO. One hundred molecular models were constructed by comparative molecular modelling through MODELLER 9.14^48^. The models were constructed using the default methods of auto-model and environ classes from MODELLER. The final models were selected according to the discrete optimized protein energy score (DOPE score). This score assesses the energy of the models and indicates the best probable structures. The best models were evaluated through PROSA II^49^ and PROCHECK^50^. PROCHECK checks the stereo-chemical quality of a protein structure through the Ramachandran plot, where good quality models are expected to have more than 90% of amino acid residues in most favored and additional allowed regions, while PROSA II indicates the fold quality. Structure visualization was done in PyMOL (http://www.pymol.org).

### Molecular Dynamics

The three-dimensional structures of clavanin A and MO were placed into a DOPC (1,2-dioleolyl-sn-glycero-3-phosphocholine) membrane using the CHARMM-GUI server^51^. The principal axis of the peptide was aligned to Z-axis and then inserted in a rectangular bilayer of 50 Å^1^ (X and Y axis) using the insertion method for system building. The system peptide-membrane was solvated with Single Point Charge water model^52^. The analyses were performed using the GROMOS96 53a6 force field extended for Berger lipids^52^ and the computational package GROMACS 4^53^. Chlorine ions were added in order to neutralize the system charge. The geometry of water molecules was constrained using the SETTLE algorithm^54^. All atom bond lengths were linked by using the LINCS algorithm^55^. Electrostatic corrections were made according to the Particle Mesh Ewald algorithm^56^, with a cut-off radius of 1.4 nm in order to minimize the computational time. The same cut-off radius was also used for van der Waals interactions. The list of neighbours of each atom was updated every 10 simulation steps of 2 fs. The system underwent an energy minimization using 50,000 steps of the steepest descent algorithm. After that, the system temperature was normalized to 310 K for 100 ps, using the velocity-rescaling thermostat (NVT ensemble). Then, the system pressure was normalized to 1 bar for 100 ps, using the Parrinello-Rahman barostat using semiisotropic pressure and the thermostat was changed to Nosé-Hoover one (NPT ensemble). The systems with minimized energy, balanced temperature and pressure were simulated for 100 ns by using the leap-frog algorithm^57^. The structures were saved every 2 ps of simulation.

### Statistical analyses

Data are presented as mean ± SD of all samples. Statistical significance of fatality rates between different groups was analysed by Kaplan–Meier test. *In vivo* assays were analysed using the Fisher´s exact test. The other data were submitted to one-way analysis of variance (ANOVA) followed by Bonferroni correction. Values of p < 0.05 were considered statistically significant. GraphPad Prism software v5.0 (GraphPad Software, USA) was used for all statistical analyses^12^.

## Additional Information

**How to cite this article**: Silva, O. N. *et al*. An anti-infective synthetic peptide with dual antimicrobial and immunomodulatory activities. *Sci. Rep*. **6**, 35465; doi: 10.1038/srep35465 (2016).

**Publisher’s note:** Springer Nature remains neutral with regard to jurisdictional claims in published maps and institutional affiliations.

## Supplementary Material

Supplementary Information

## Figures and Tables

**Table 1:** Antibacterial activity of clavanin A and synthetic peptide clavanin-MO.

Microorganisms	MIC (μM)				
	Clavanin A	Clavanin-MO	LL-37	Gentamicin	Imipenem
**Gram-positive**					
*B. subtilis* ATCC6633	6.0	1.5	6.0	6.0	0.5
*E. faecalis* ATCC12953	6.0	1.5	6.0	6.0	0.5
*S. aureus* ATCC29213	48.0	24.0	12.0	1.5	0.5
*S. aureus* (MRSA) ATCC33591	12.0	6.0	48.0	48.0	12.0
*S. pyogenes* ATCC19615	12.0	6.0	24.0	24.0	0.5
**Gram-negative**					
*E. coli* ATCC8739	24.0	12.0	12.0	0.5	1.5
*E. coli* KPC-positive (1812446)	48.0	6.0	24.0	6.0	48.0
*E. coli* (2101123)	12.0	6.0	24.0	48.0	48.0
*K. pneumoniae* ATCC13885	6.0	3.0	3.0	1.5	1.5
*K. pneumoniae* 1825971 (KPC971)	6.0	3.0	12.5	6.0	48.0
*P. mirabilis* ATCC25933	12.0	6.0	6.0	6.0	3.0
*P. aeruginosa* ATCC 15442	12.0	3.0	12.5	1.5	1.5
*S. enterica* ATCC14028	6.0	3.0	6.0	6.0	3.0

Bacteria were cultured in the presence of different peptide concentrations. Gentamicin and human cathelicidin peptide LL-37 were used as positive controls. Data are representative of three independent experiments.
