# Supplementary material for: An anti-infective synthetic peptide with dual antimicrobial and immunomodulatory activities
Source: Sci Rep. 2016 Nov 2;6:35465. doi: 10.1038/srep35465 (PMC5090204; doi:10.1038/srep35465)
Supplement: Supplementary Information [file srep35465-s1.doc]

**Supplemental Information**

**An anti-infective synthetic peptide with dual antimicrobial and immunomodulatory activities**

O. N. Silva^1,2#^, C. de la Fuente-Núñez^3-8#^, E. F. Haney^9#^, I. C.M. Fensterseifer^10,11^, S. M. Ribeiro^2^, W. F. Porto^10^, P. Brown^12^, C. Faria-Junior^13^, T.M.B. Rezende^10,14,15^, S. E. Moreno^2^, T. K. Lu^3-8^, R. E. W. Hancock^9^ and O. L. Franco^1,2,10,11,*^

# Table S1. Amino acid sequences of peptides used in this study.

| **Peptide** | **Sequence** | **Length** |
| --- | --- | --- |
| Clavanin A | VFQFLGKIIHHVGNFVHGFSHVF-NH_2_ | 23 |
| Clavanin-MO | FLPIIVFQFLGKIIHHVGNFVHGFSHVF-NH_2_ | 28 |
| LL-37 | LLGDFFRKSKEKIGKEFKRIVQRIKDFLRNLVPRTES-NH_2_ | 37 |

**Figure S1. Peptide activity against flow cell-grown biofilms.** Anti-biofilm activity of clavanin-MO against KPC producing *Klebsiella* and methicillin-resistant *S. aureus* strains. Clavanin-MO repressed *Klebsiella* preformed biofilms. On the other hand, Clavanin-MO had virtually no inhibitory effect on preformed MRSA biofilms at the concentrations tested.

**
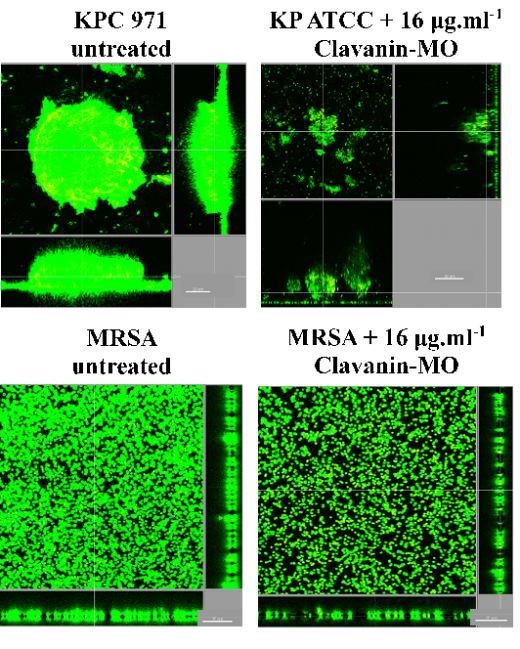
**

**Figure S2. Peptides acted through a stress response-independent pathway.** Neither clavanin A nor clavanin-MO interfered with cellular levels of ppGpp as determined by thin layer chromatography separation of guanine nucleotides extracted from live *P. aeruginosa* cells.

**
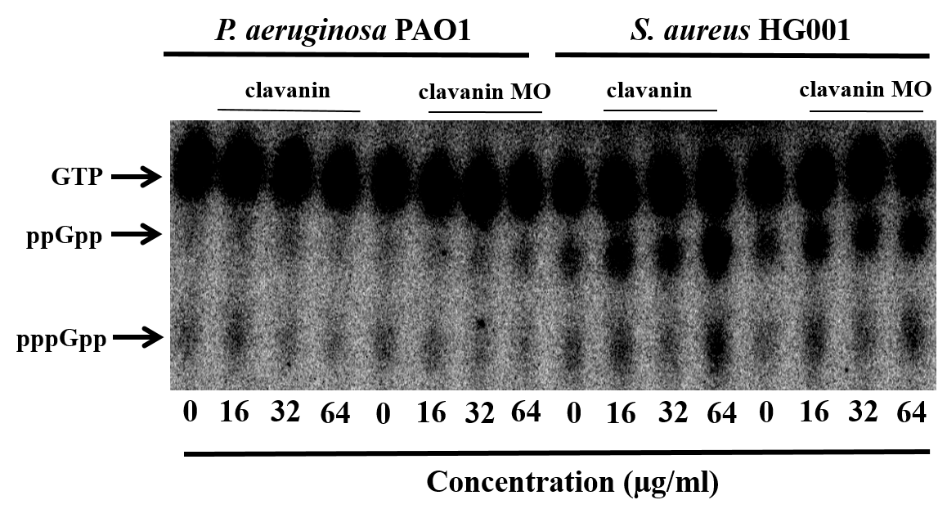
**

**Figure S3. Peptide structural characterization. (A) Initial and final structures of clavanin A (upper panels) and clavanin-MO (bottom panels) embedded in a DOPC bilayer.** The structures are in the same orientation. The clavanin-MO model has a DOPE score of -2130.25; a Z-score on ProSa of 1.15; 100% of the residues in favoured regions of the Ramachandran plot and an overall G-factor of -0.02. All validations indicated that this is a valid model. The simulations of peptides embedded in the membrane showed that, in both cases, only the peptide orientation along the Z-axis is changed in relation to the initial position. **(B)** **The backbone’s RMSD evolution during the simulation.** The black line indicates the clavanin A simulation, while the red one, the clavanin-MO. During the simulation, the RMSD was below 4 Å, indicating that the α-helical structure is maintained.

**
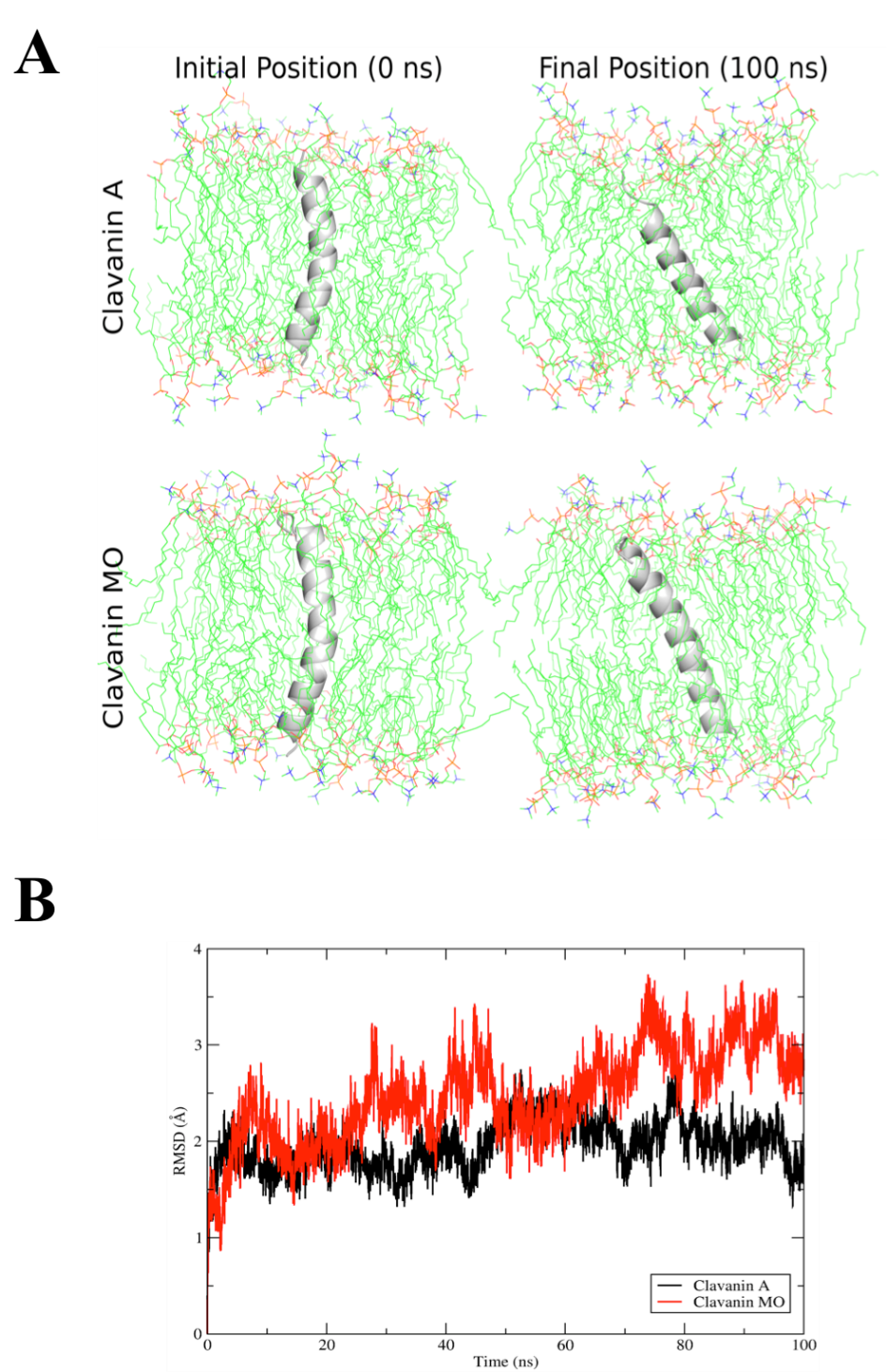
**
